# Maternal Mortality Over a Seven-Year Period of Conflict at Jiblah Referral Hospital in Ibb City, Yemen: A Retrospective Observational Study

**DOI:** 10.7759/cureus.41044

**Published:** 2023-06-27

**Authors:** Afaf Alsharif, Faisal Ahmed, Abdullah M Almatary, Mohamed A Badheeb

**Affiliations:** 1 Department of Gynaecology, Jiblah University for Medical and Health Sciences, Ibb, YEM; 2 Department of Urology, Ibb University, Ibb, YEM; 3 Department of General Surgery, Jiblah University for Medical and Health Sciences, Ibb, YEM; 4 Department of Internal Medicine, Hadhramout University, Hadhramaut, YEM

**Keywords:** yemen, health facility, ibb, maternal death, conflict

## Abstract

Background

Fragile and conflict-affected states contribute to more than 60% of the global burden of maternal mortality. There is an alarming need for research exploring maternal health service access, quality, and adaptive responses during armed conflict. This study aims to review all cases of maternal mortality during a seven-year period of conflict at Jiblah Referral Hospital, Ibb, Yemen.

Methodology

A retrospective, observational study was conducted between 2011 and 2017, including all maternal deaths that occurred at Jiblah Referral Hospital, Ibb, Yemen. Data on maternal demographics, characteristics, intrapartum care, and cause of death were collected. Additionally, we compared patient characteristics according to residency (rural versus urban).

Results

During the study period, of the 2,803 pregnant women admitted to our hospital, 52 maternal deaths occurred. Their mean age was 29.0 ± 6.2 years, and most (63.5%) were aged less than 30 years. Most (88.5%) did not have a regular antenatal care visit, were referred cases (86.5%), were residents of rural areas (63.5%), and had a low socioeconomic condition (59.6%). The majority of maternal deaths were reported among women with gestational age (GA) of 24-34 weeks (57.7%) and primiparas women (42.3%). At hospital arrival, the majority of cases were in shock (69.2%). The majority of the mothers died during the intrapartum period (46.2%). The main cause of death was severe bleeding (32.7%), followed by eclampsia (25.0%). The mean time from admission to death was 3.0 ± 1.2 days (range = 1-6). Among all maternal deaths, 76.9%, 75.0%, and 26.9% had delays in seeking care, delays in reaching first-level health facilities, and delays in receiving adequate care in a facility, respectively. Additionally, most patients had at least two delays (57.7%). These delays were due to unawareness of danger signs in 57.7% and illiteracy and ignorance in 78.8% of cases. In comparison, according to residency, maternal mortality was statistically significant among mothers living in a rural area with GA of 25-34 weeks (24 vs. 6, p = 0.015). Additionally, maternal mortality due to delay in seeking care, unawareness of danger signs, and having at least two delays were statistically significant among rural mothers (p < 0.05).

Conclusions

Our study demonstrates that maternal deaths occurred among young women, referred cases, with no regular antenatal care visits, low socioeconomic conditions, and who were residents of rural areas. Delays in seeking care and delays in reaching first-level health facilities were the most common causes of maternal death due to unawareness of danger signs, illiteracy, and ignorance. We recommend that imparting basic skills and improving awareness in the community about the danger signs of pregnancy can be effective measures to detect maternal complications at an earlier stage, especially in rural areas.

## Introduction

According to the World Health Organization (WHO), in 2020, over 800 women died every day during pregnancy or from pregnancy-related problems [1,2]. Low and lower-middle-income countries accounted for over 95% of the global maternal mortality burden [2]. While a survey mentioned an improvement in maternal mortality in Yemen (68.0%) from 1990 to 2017, recent data in 2023 mentioned that Yemen still has one of the highest maternal mortality rates according to the Fragile States Index [3]. The current conflict in Yemen has displaced millions and destroyed health infrastructure, resulting in the world’s largest humanitarian disaster. According to a recent survey in Yemen conducted between 2012 and 2019, an estimated 168,212 excess deaths occurred between 2015 and 2019 and a 17.8% increase in overall deaths, and Ibb governorates presented one of the highest total excess deaths [4]. According to a recent study, the ongoing Yemen catastrophe is underappreciated and mostly ignored in comparison to this high number [5]. Surprisingly, there has been a significant underestimation of the scope of prenatal disorders and their relationship with both perinatal and early neonatal mortality in underdeveloped nations. Because of the prevalence of unregistered births, policymakers are unaware of the magnitude of perinatal fatalities. Yemen’s perinatal circumstances are proven to be a serious public health issue [6]. Yemeni women face a 1 in 90 lifetime risk of maternal death. United Nations International Children’s Emergency Fund estimates that 470 women per 100,000 live births die from obstetric complications. An estimated 82% of these deaths occur during delivery [2].

In recent research done in Sana’a City between 2015 and 2016, 952 pregnant women were followed up for up to seven days after giving birth. The rates of prenatal death, stillbirth, and early neonatal mortality were 89.3 per 1,000, 46.2 per 1,000, and 45.2 per 1,000, respectively [6]. As a result, pregnancy-related problems continue to have a significant impact on the lives of mothers and their infants. To meet the development target for maternal and child health, increased access and coverage of important interventions, as well as improvements in care quality, are required [7]. Maternal death reviews at healthcare institutions, also known as maternal death audits, aid in understanding the importance of quality of care. These audits discover obstetric causes of maternal mortality and give thorough information on preventable maternal mortality factors [8]. The analysis of these fatalities using a facility-based maternal death review technique also provides a clear picture of the various forms of delays that contribute to maternal deaths at various stages [9]. This study aims to review the causes of maternal deaths and their associated factors in Jiblah Referral Hospital, Ibb, Yemen during a period of conflict between 2011 and 2017 and discuss the remedy.

## Materials and methods

Study settings

A retrospective, observational study was conducted between 2011 and 2017, including all maternal deaths that occurred at Jiblah Referral Hospital, Ibb, Yemen. Data on maternal demographics, characteristics, intrapartum care, birth outcomes, and cause of death were collected. The hospital’s maternal mortality registry was used to identify all maternal deaths that occurred during the study period. The Ethics Research Committees of Jiblah University for Medical Sciences provided approval for the study on 01.05.2023. This study adhered to the ethical principles outlined in the Declaration of Helsinki.

Data collection and definition

We collected data regarding age, residency, antenatal care, referral status, socioeconomic condition, gestational age, gravidity, parity, previous abortions, condition at the time of admission, the time between admission to death, the mode of delivery, fetal malformation, and cause of maternal death. Additionally, data on the type of delay and ignorance, such as delay in seeking care, unawareness of danger signs, illiteracy and ignorance, delay in reaching first-level health facilities, delay in receiving adequate care in a facility, and lack of blood equipment or drug, were collected.

Maternal deaths were defined as deaths among reproductive-aged women (15-49 years) caused by pregnancy-related problems and childbirth that occurred within 42 days following delivery [9]. For collecting relevant data, the maternal death review forms and bedhead tickets were employed. In this study, the “Three Phases of Delay Model” was used to categorize variables related to maternal deaths. Type I delay refers to a delay in seeking modern medical care, type II delay refers to a delay in reaching a health facility, and type III delay refers to a delay in receiving appropriate care at the health facility [9]. We do not routinely monitor thromboprophylaxis in our hospital as most pregnant mothers arrive underweight.

Study objective

To report the maternal mortality and compare the patient characteristics according to residency (rural versus urban).

Statistical analysis

Quantitative data were presented as means and standard deviations, while qualitative variables were reported as frequencies and percentages. The Smirnov-Kolmogorov test confirmed the normality of the data. For quantitative variables, the independent-samples t-test or Mann-Whitney test was used. The chi-square or Fisher’s exact test was used to compare qualitative variables. A p-value <0.05 was considered statistically significant. Statistical analysis was performed using SPSS version 22 software (IBM Corp., Armonk, NY, USA).

## Results

Patients’ characteristics

During the study period, of the 2,803 pregnant women admitted to our hospital, 52 maternal deaths occurred. Their mean age was 29.0 ± 6.2 years (range = 16-41), and most (63.5%) were aged less than 30 years. Most (88.5%) did not have a regular antenatal care visit. Most mothers were referral cases (86.5%), residents of rural areas (63.5%), and had a low socioeconomic condition (59.6%). The demographic characteristics of maternal death patients are shown in Table 1.

**Table 1:** Demographic characteristics of maternal death patients.

Factors	N (%)
Age (year), mean ± SD	29.0 ± 6.2 (range = 16–41)
Age groups	
<20 years	5 (9.7%)
20–30 years	28 (53.8%)
≥30 years	19 (36.5%)
Antenatal care	
No	46 (88.5%)
Yes	6 (11.5%)
Referral status	
No	7 (13.5%)
Yes	45 (86.5%)
Socioeconomic condition	
High	8 (15.4%)
Medium	13 (25.0%)
Low	31 (59.6%)
Residency	
Urban	19 (36.5%)
Rural	33 (63.5%)
Malformation of baby	11 (21.2%)

**Figure 1:**
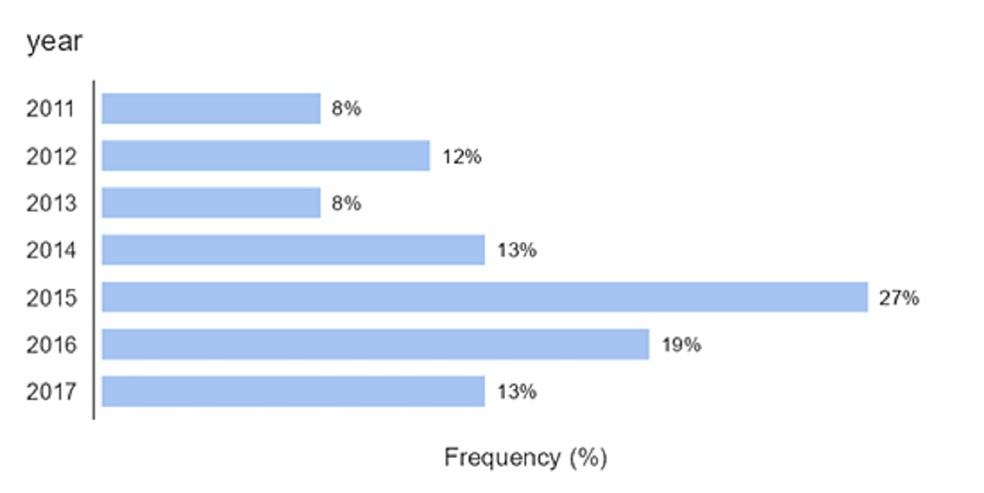
Percentage distribution of maternal deaths according to the year of admission (n = 52).

Between 2011 and 2017, the most maternal mortality occurred in 2015 (27%) (Figure 1).

Pregnancy characteristics and causes of maternal death

The majority of maternal deaths were reported at gestational age (GA) of 24-34 weeks (57.7%) in multigravida (63.5%) and primiparas women (42.3%). Overall, 46.2% of mothers had at least one abortion. At hospital arrival, the majority of cases were in shock (69.2%). The majority of the mothers died during the intrapartum period (46.2%). The main cause of death was severe bleeding (32.7%), followed by eclampsia (25.0%), and obstructed labor/ruptured uterus (17.3%). The mean time from admission to death was 3.0 ± 1.2 days (range = 1-6 days). The majority of deaths (75.0%) occurred within three days of admission. Pregnancy characteristics and causes of maternal deaths are shown in Table 2.

**Table 2:** Pregnancy characteristics and causes of maternal deaths. IUFD: intrauterine fetal death; DIC: disseminated intravascular coagulation

Factors	N (%)
Gestational age (weeks), mean ± SD	31.7 ± 5.8 (range = 20–41)
Gestational age groups	
<24 weeks	2 (3.8%)
24–34 weeks	30 (57.7%)
≥34 weeks	20 (38.5%)
Gravidity, mean ± SD	4.3 ± 3.5 (range = 1–12)
Gravidity group	
Primigravid	19 (36.5%)
Multigravida	33 (63.5%)
Parity, mean ± SD	2.9 ± 2.9 (range = 0–10)
Parity group	
Primiparous	22 (42.3%)
Parity of 1–4	17 (32.7%)
Parity ≥5	13 (25.0%)
Previous abortions	24 (46.2%)
Period of death	
Antenatal before 20 weeks	2 (3.8%)
Antenatal after 20 weeks	11 (21.2%)
Postpartum/Postnatal	24 (46.2%)
Intrapartum	15 (28.8%)
Condition at admission	
Not stable or in shock	36 (69.2%)
Semi-stable	16 (30.8%)
The time between admission to death (day), mean ± SD	3.0 ± 1.2 (range = 1–6)
Time death group	
Less than 3 days	39 (75.0%)
More than 3 days	13 (25.0%)
Cause of death	
Severe bleeding (postpartum hemorrhage)	17 (32.7%)
Eclampsia	13 (25.0%)
Obstructed labor/Ruptured uterus	9 (17.3%)
Infections/Sepsis	3 (5.8%)
IUFD with DIC	4 (7.7%)
Ectopic pregnancy	3 (5.8%)
Heart failure	2 (3.8%)
Other	1 (1.9%)
Mode of delivery	
Cesarean section	7 (13.5%)
Normal vaginal delivery	45 (86.5%)

Maternal death according to the type of delay and ignorance

Among all maternal deaths, 76.9%, 75.0%, and 26.9% had delays in seeking care, delays in reaching first-level health facilities, and delays in receiving adequate care in a facility, respectively. Additionally, most patients had at least two delays (57.7%). These delays were due to unawareness of danger signs in 57.7% and illiteracy and ignorance in 78.8% of cases (Table 3).

**Table 3:** Distribution of maternal deaths according to the type of delay and ignorance.

Factors	N (%)
Delay in seeking care	
No	12 (23.1%)
Yes	40 (76.9%)
Unawareness of danger signs	
Yes	30 (57.7%)
No	22 (42.3%)
Illiteracy and ignorance	
No	11 (21.2%)
Yes	41 (78.8%)
Delay in reaching first-level health facilities	
No	13 (25.0%)
Yes	39 (75.0%)
Delay in receiving adequate care in a facility	
No	38 (73.1%)
Yes	14 (26.9%)
Lack of blood equipment or drug	
No	44 (84.6%)
Yes	8 (15.4%)
Total number of delays	
No delay	3 (5.8%)
One delay	12 (23.1%)
Two delays	30 (57.7%)
Three delays	7 (13.5%)

Comparison of maternal mortality in urban and rural cases

Maternal mortality was statistically significant among mothers living in a rural area with GA of 25-34 weeks (24 vs. 6, p = 0.015). Additionally, maternal mortality due to delay in seeking care, unawareness of danger signs, and having at least two delays were statistically significant among mothers living in rural areas (all p < 0.05) (Table 4).

**Table 4:** Comparison of maternal deaths according to residency. IUFD: intrauterine fetal death; DIC: disseminated intravascular coagulation

Factors	Urban (N = 19)	Rural (N = 33)	P-value
Age (year), mean ± SD	29.2 ± 6.3	28.9 ± 6.2	0.904
Antenatal care			0.467
No	16.0 (84.2%)	30.0 (90.9%)	
Yes	3.0 (15.8%)	3.0 (9.1%)	
Referral status			0.638
No	2.0 (10.5%)	5.0 (15.2%)	
Yes	17.0 (89.5%)	28.0 (84.8%)	
Socioeconomic condition			0.661
High	4.0 (21.1%)	4.0 (12.1%)	
Medium	4.0 (21.1%)	9.0 (27.3%)	
Low	11.0 (57.9%)	20.0 (60.6%)	
Mode of delivery			0.638
Cesarean section	2.0 (10.5%)	5.0 (15.2%)	
Normal vaginal delivery	17.0 (89.5%)	28.0 (84.8%)	
Malformation of baby			0.989
No	15.0 (78.9%)	26.0 (78.8%)	
Yes	4.0 (21.1%)	7.0 (21.2%)	
Gestational age groups			0.015
<24 weeks	1.0 (5.3%)	1.0 (3.0%)	
24–34 weeks	6.0 (31.6%)	24.0 (72.7%)	
≥34 weeks	12.0 (63.2%)	8.0 (24.2%)	
Gravidity group			0.573
Primigravid	6.0 (31.6%)	13.0 (39.4%)	
Multigravida	13.0 (68.4%)	20.0 (60.6%)	
Parity group			
Primiparous	8.0 (42.1%)	14.0 (42.4%)	
Parity of 1–4	6.0 (31.6%)	11.0 (33.3%)	
Parity ≥5	5.0 (26.3%)	8.0 (24.2%)	
Previous abortions			0.894
No	10.0 (52.6%)	18.0 (54.5%)	
Yes	9.0 (47.4%)	15.0 (45.5%)	
Cause of death			0.243
Eclampsia	4.0 (21.1%)	9.0 (27.3%)	
Severe bleeding	5.0 (26.3%)	12.0 (36.4%)	
Infections/Sepsis	0.0 (0.0%)	3.0 (9.1%)	
Obstructed labor/Ruptured uterus	5.0 (26.3%)	4.0 (12.1%)	
Ectopic pregnancy	2.0 (10.5%)	1.0 (3.0%)	
IUFD with DIC	3.0 (15.8%)	1.0 (3.0%)	
Heart failure	0.0 (0.0%)	2.0 (6.1%)	
Other	0.0 (0.0%)	1.0 (3.0%)	
Period of death			0.759
Antenatal before 20 weeks	1.0 (5.3%)	1.0 (3.0%)	
Antenatal after 20 weeks	5.0 (26.3%)	6.0 (18.2%)	
Postpartum/Postnatal	7.0 (36.8%)	17.0 (51.5%)	
Intrapartum	6.0 (31.6%)	9.0 (27.3%)	
Condition at admission			0.076
Not stable or in shock	16.0 (84.2%)	20.0 (60.6%)	
Semi-stable	3.0 (15.8%)	13.0 (39.4%)	
Delay in seeking care			<0.001
No	10.0 (52.6%)	2.0 (6.1%)	
Yes	9.0 (47.4%)	31.0 (93.9%)	
Unawareness of danger signs			<0.001
Yes	17.0 (89.5%)	13.0 (39.4%)	
No	2.0 (10.5%)	20.0 (60.6%)	
Illiteracy and ignorance			0.162
No	6.0 (31.6%)	5.0 (15.2%)	
Yes	13.0 (68.4%)	28.0 (84.8%)	
Delay in reaching first-level health facilities			0.868
No	5.0 (26.3%)	8.0 (24.2%)	
Yes	14.0 (73.7%)	25.0 (75.8%)	
Delay in receiving adequate care in a facility			0.469
No	15.0 (78.9%)	23.0 (69.7%)	
Yes	4.0 (21.1%)	10.0 (30.3%)	
Lack of blood equipment and drug			0.390
No	15.0 (78.9%)	29.0 (87.9%)	
Yes	4.0 (21.1%)	4.0 (12.1%)	
The time between admission to death (day), mean (SD)	2.8 (1.3)	3.0 (1.2)	0.587
Any delay			0.035

## Discussion

Currently, our understanding of maternal mortality and its associated factors in developing countries is very poor, partly due to the scarcity of data related to maternal deaths and their determinants [4]. Examining the enormity of the issue, we tried to explore the relationship between maternal death and associated factors. This attempt was made using the facility-based maternal death review approach for determining the causes of maternal deaths and their circumstances in the Ibb governorate.

Yemen has one of the highest maternal mortality rates in the world, a situation that has deteriorated since 2014 because of the war. In 2019, in Yemen, one woman and six newborns die every two hours because of complications during pregnancy or birth, with a mortality rate of 164 per 100,000 live births [10].

In this study, the majority of maternal mortality occurred in the 20-30-year age group. This is similar to previous reports such as the study reported by Sk et al. in West Bengal [9]. Additionally, in another study by Al-Shahethi et al. in Sana’a City, Yemen, between 2015 and 2016, 85.3% of maternal mortality cases were aged between 18 and 34 years [6]. This is concomitant with the prevailing custom of early marriage in our city.

In this study, maternal mortality was seen among multigravida, primiparous, and those with a history of previous abortions. Our findings were similar to previous reports in our country [6].

Nearly 75% of deaths were due to preventable causes, including severe hemorrhage, pre-eclampsia/eclampsia, infection, complications during delivery, and unsafe abortions [11]. Similarly, in our study, the most common cause of maternal mortality was severe bleeding, followed by hypertensive disorders of pregnancy or eclampsia and obstructed labor/ruptured uterus. Further efforts are needed to improve the availability and quality of data related to maternal mortality.

In this study, the majority of maternal mortality occurred in poor households. This is similar to previous reports by Alam et al. and Kingsley et al. [12-14]. One of the reasons for the high maternal mortality rate in poor households is the inability to access maternal and reproductive health services, as well as the lack of transport to healthcare services.

In this study, maternal mortality was higher in those living in rural areas, with low socioeconomic conditions, and those with no regular antenatal visits. These factors are essential and were investigated in previous studies [6]. For example, Butt et al. mentioned the factors contributing to the many crises in Yemen which led to substantially high rates of maternal mortality [10]. The study mentioned that instead of ongoing war and the COVID-19 pandemic, food insecurity, poverty, pollution, illiteracy, and lack of access to basic major resources affected the maternal mortality rate [10]. Additionally, studies are consistent in their findings about the dissatisfaction with government services, including rude and improper behavior by the health staff, staff shortages, and lack of supplies and drugs. Distance to healthcare services, time, charges, and the behavior of providers play a major role in making the urban poor’s decision to seek healthcare services [6,15]. Additionally, unlike the rural poor, the urban poor have a variety of healthcare services available to them in the city. The services are available at tertiary-level teaching hospitals and ordinary dispensary services [15].

An overwhelming majority of maternal mortality in this study (86.5%) was seen among referral cases; most of such referrals were from subdivision hospitals/rural hospitals or community health centers and were in critical or irreversible condition at the time of admission. Additionally, 46.2% died within the postpartum period. Our finding was similar to previous studies by Sk et al. and Bhadra et al. [9,16].

It is important to investigate the delays at various stages and ignorance leading to deaths. In this study, among all maternal deaths, 76.9%, 75.0%, and 26.9% had delays in seeking care, delays in reaching first-level health facilities, and delays in receiving adequate care in a facility, respectively. Additionally, most patients had at least two delays (57.7%). These delays were due to unawareness of danger signs in 57.7% and illiteracy and ignorance in 78.8% of cases. In comparison, according to residency, maternal mortality was statistically significant among mothers living in a rural area with GA of 25-34 weeks (24 vs. 6, p = 0.015). Additionally, maternal mortality due to delay in seeking care, unawareness of danger signs, and having at least two delays were statistically significant among rural mothers (p < 0.05). This was similar to several previous reports [9,16-19]. The population growth in slum areas, tradition, lack of education, and limited budgets may be crucial factors in deterring women from using health services [6]. In a study by Sitaula et al., the primary contributory factors of maternal mortality were delays in seeking healthcare and reaching healthcare facilities [20]. Training of medical officers and staff nurses working in rural areas through programs such as basic emergency obstetrics care and the presence of skilled attendants at birth training give a ray of hope for reducing maternal mortality. Maternal deaths can be prevented by improving the healthcare facilities in rural areas by ensuring continuous availability of basic drugs such as injection magnesium sulfate and tablet misoprostol as most maternal deaths in rural areas are still due to eclampsia and postpartum hemorrhage [16,21]. Blood banks should also be available in rural health centers. The provision of 24-hour ambulance services for the transportation of patients should be seriously considered. Roads in rural areas should be well connected to the nearby towns. Proper health education should be started in schools to reduce the incidence of mortality [16]. Hyzam et al. studied health information and health-seeking behavior in Yemen in rural areas [22]. They reported that health education and promotion activities on maternal health were ad hoc and coverage was poor. Maternal health services were underutilized by women. According to the data from the focus group discussions, the poor quality of services, as indicated by the inadequate number of female doctors, lack of medical equipment and medicines, and costs of services were barriers to the use of maternal health services. Moreover, the use of prenatal and postnatal care services was associated with women’s perceived needs. However, according to health professionals, inadequate human resources, workload, and inadequate funding from the government have contributed significantly to the perceived quality of maternal health services provided by public health facilities. Despite the identified barriers, we found that a safe motherhood voucher scheme was instituted in Lahj which facilitated the use of maternal health services for disadvantaged women by removing financial barriers associated with the use of maternal health services [22].

This study had several limitations. Its retrospective and monocentric design was the main limitation. Hence, the generalization of our findings should be done with caution. Second, some of the deaths that occurred at home did not receive death certificates and were not recorded in any formal system. Therefore, many of these deaths would have been missed by facility-based studies. Third, several factors such as khat chewing, smoking, education level, government policies, patient ethics, nutrition status, presence of other comorbidities, and the role of support by several health organizations were not investigated. In fact, with the support of WHO and other organizations in facilitating and funding our hospital, the rate of admission and offering several maternal services had a role in the improvement in our hospital which will be investigated in the future. Finally, the neonatal mortality rate and its associated factors were not analyzed.

## Conclusions

Our study demonstrates that maternal deaths occurred among young women, referred cases, those with no regular antenatal care visits, those with low socioeconomic conditions, and who were residents of rural areas. Delays in seeking care and delays in reaching first-level health facilities were the most common causes of maternal death due to unawareness of danger signs, illiteracy, and ignorance. We recommend that imparting basic skills and improving awareness in the community about the danger signs of pregnancy can be effective measures to detect maternal complications at an earlier stage, especially in rural areas. Further efforts are needed to manage and solve the underlying causes alongside focusing on the ways to decrease maternal mortality.
